# Factors influencing enrolled nursing students’ academic performance at a selected private nursing education institution in KwaZulu-Natal

**DOI:** 10.4102/curationis.v41i1.1850

**Published:** 2018-08-28

**Authors:** Makhosazane B. Dube, Puseletso R. Mlotshwa

**Affiliations:** 1 School of Nursing and Public Health, University of KwaZulu-Natal, South Africa

## Abstract

**Background:**

The success of any educational institution is measured by its academic performance or how well students meet the standards set out. Currently, nursing students’ academic failure is a phenomenon of growing international interest because of its economic impact and its negative effects on the availability of future nurses in different health care systems. Factors identified as influencing the academic performance of students include the socio-economic background of parents or guardians, lecturer–student relationships, academic support services, demographic factors, quality of nurse educators, availability of facilities in the school, the language of instruction and level of entry qualifications of students.

**Objectives:**

The purpose of this study was to describe the perceptions of enrolled student nurses on factors influencing their academic performance in a private nursing school in KwaZulu-Natal, South Africa.

**Method:**

Data were collected from 100 respondents using an adapted instrument related to the factors believed to influence students’ academic performance.

**Results:**

The results showed that parental involvement in education, good and supportive relationships between nurse educators and students, classroom computer technological gadgets, internet connection and adequate learning facilities were perceived as fostering better academic performance of students. In contrast, poor family background, use of English language for classroom instruction as well as negative peer group influences were leading to poor academic performance.

**Conclusion:**

Nursing institutions should, therefore, select students with higher-level entry qualifications, early identify at-risk students, recruit more qualified nurse educators and upgrade their facilities.

## Introduction

In every educational institution, success is measured either by the academic performance of the students (Ushie et al. 2012:178) or by how well students meet the standards set out by the local government and the institution itself (Bell 2011). In nursing, factors such as the terminology, the academic workload, and the responsibility or human life in the clinical field place enormous stress on the student and can affect their academic performance (Alden 2008:63). In addition, factors such as the socio-economic background, the ability of students to handle the change from a school teaching environment to a nursing college learning environment and low self-efficacy contribute to the academic performance of enrolled nursing students (Jafta 2013:2).

## Literature review

Education is a consistently important national issue that frequently brings focus to bear on stakeholders in the education industry (Dimkpa & Inegbu 2013:2). Clearly too, the effort and resources that go into recruiting students will have little value if educative resources and curricula fail to prepare the students for academic success (Blackman, Hall & Darmawan 2007:222). Academic performance involves the ability of students to cope with their studies with the various tasks assigned to them by their instructors (MacFarlane 2002, cited in Dimkpa & Inegbu 2013:2). It appears to be a problem at all academic institution, not just nursing colleges (Jafta 2013:1). Nurse training institutions are a significant part of the South African educational landscape, playing an important role in preparing student nurses for professional competence in the field (Dimkpa & Inegbu 2013:1). Therefore, academic failure in colleges of nursing would not only frustrate the students and parents but also have a devastating effect on society in terms of workforce shortage in the nation’s health sector (Aremu 2000, cited in Dimkpa & Inegbu 2013:2).

Numerous factors affect the academic progress and learning performance of students: age (Blackman et al. 2007:232), gender, school education, residential area students come from, medium of instruction in schools, tuition trends, daily study hours, accommodation and the socio-economic background of the parents or guardians (Ali et al. 2013:284).

According to available literature, there are some impediments to good academic performance by student nurses. They include excessive homework assignments for the students, poor facilities, inadequate provision of basic needs by parents and inappropriate student perceptions (Dimkpa & Inegbu 2013:4). Poor teaching (Manson 2014:37), misunderstanding of academic questions (Bradbury & Miller 2011:117–118) and time constraints when students have to read the question, translate it into the home language and then choose the correct answer (Doley 2010:1809) are some more.

In contrast, social and emotional lecturer–student relationships (Creasey et al. 2009 cited in Nyadanu et al. 2015:266), a cohesive school atmosphere (Chowa et al. 2015:132), a welcoming atmosphere (Courtney-Pratt et al. 2011) and students with higher-level entry qualifications (McCarey, Barr & Rattray 2007) are some predictors of students’ good academic performance. Moreover, a proper clinical education and an appropriate learning environment (Severinsson & Sand 2010) can lead to an adequate clinical performance.

## Method

A quantitative design, namely a survey, was used to determine the perceptions of enrolled student nurses on factors influencing their academic performance in a private nursing school in KwaZulu-Natal, South Africa.

### Study respondents and setting

The target population was enrolled student nurses in nursing schools in KwaZulu-Natal. Respondents included first- and second-year enrolled nursing students at a nursing training institution who had registered for the nursing course with the South African Nursing Council. A simple random sampling was decided upon. A sample frame with a list of students’ names according to their year of study was used to select the sample randomly. The sample size was calculated using a computer program known as Raosoft Sample Calculator, employing the following parameters to ensure representativeness: margin of error of 5%, confidence level of 95%, a response rate of 50% and a population of 120 as sample size. The setting was selected for its easy access by the researcher.

### Data collection

The instrument for data collection was adopted from Mushtaq and Khan (2012:19) and Kyoshaba (2009:23), related to the factors believed to influence students’ academic performance. The major concepts in this framework were (1) communication, (2) learning facilities, (3) proper guidance and (4) family stress. The researcher’s contribution to this study was to describe the four factors believed to influence students’ academic performance. It was hypothesised that the academic performance of students would be improved if the four factors (communication skills, learning facilities, proper guidance and family stress) were properly addressed and monitored.

Respondents were requested to sign a declaration form as soon as they agreed to participate in the study. The researcher explained to them how to complete the questionnaire to avoid wastage, errors and spoiled questionnaires. Thereafter, the questionnaires were distributed by the researcher herself to the students in the classroom at the school twice over a period of 1 week to a group of 60 students. Only 100 were returned out of the 120 questionnaires distributed.

### Measurements

The researcher measured the perceptions of the students related to communication, the facilities, proper guidance and family stress. The respondents were asked to agree or not to a list of statements to measure their perceptions, on a five-point Likert scale.

### Data analysis

The data were captured on spreadsheets after having been gathered and examined for completeness. The questionnaires were coded, computed and analysed using the Statistical Package for Social Sciences (SPSS), Version 23.0, with the help of the University KwaZulu-Natal statistical consultation service. Descriptive statistics were used to summarise and analyse data using frequency tables, graphs and charts, as these provide an accurate and clear picture of the results for easy understanding.

### Ethical considerations

The researcher obtained the permission to conduct this study from the nursing school and ethical approval (HSS/1568/015M) from the University of KwaZulu-Natal prior to data collection.

## Results

This section displays the demographic details as well as the perceived factors that influence students’ academic performance.

### Demographic details

Of the 100 students, 91.0% *(n* = 91) were above 20 years of age, 80.0% (*n* = 80) were female and 93.0% (*n* = 93) were isiZulu-speaking. The findings showed that out of the 16% (*n *= 16) who had repeated a grade in high school, 14% (*n* = 14) of total respondents had repeated 1 year and 2% (*n* = 2) had repeated 2 years.

### Communication

Eight items helped to determine the student nurses’ perceptions of factors relating to communication skills that influenced their academic performance (Table 1). These results suggest empirically that the use of information communication technology (ICT) has a positive effect on academic performance of student nurses. The results showed that phone calls, emails and electronic discussion boards classroom computer technological gadgets and internet services were reported to have a positive influence on the academic performance of 74% (*n* = 74), 80% (*n* = 80) and 70% (*n* = 70) of the respondents, respectively. The youth of today are technology users and they prefer using technology for learning and interaction (Manson 2014:102). E-learning makes it easy to perform activities such as uploading educational materials and conducting online assignments and continuous assessment (Paraia, Shenoya & Oh 2013:80). It also improves students’ interest and ability in the classroom, such that students’ retention, motivation and class participation are increased (Simpson & Courtney 2008:637). Yet, the tendency to chat with friends and relatives via WhatsApp has been reported by 63% (*n* = 63) of the respondents.

**TABLE 1:** Student nurses’ perceptions of factors relating to communication skills that influenced their academic performance.

Factors relating to communication skills that influenced the students’ academic performance	Disagree		Neutral		Agree	
	n	%	n	%	n	%
Communication through phone calls, emails, and electronic discussion boards facilitates easy transmission of information between the teacher and the learner.	74	74	12	12	14	14
The classroom computer technological gadgets are important tools to engage student nurses in meaningful learning.	80	80	6	6	7	7
The school internet and my phone internet service favour my academic performance.	70	70	21	21	8	8
I spend time searching for study materials and chatting with friends and relatives via WhatsApp.	63	63	18	18	19	19
Nurse educator–student relationships play a meaningful role in my schooling and academic achievements (learning gains).	85	85	8	8	6	6
A good and supportive relationship between nurse educators and students fosters better academic performance of students.	92	92	4	4	4	4
Influences from peer group cause poor academic performance of students.	67	67	13	13	18	18
The use of the English language is a barrier to my learning as it affects my understanding of the topics discussed in class.	69	69	7	7	23	23

*n*, number of respondents.

For 85% (*n* = 85) of the respondents, nurse educator–student relationships played a meaningful role in their academic achievements. Similarly, 92% (*n* = 92) of the respondents reported that a good and supportive relationship between nurse educators and students fostered better academic performance. Undoubtedly, instructor–student relationships are essential to one’s social and emotional development; they have the potential to influence how a student will succeed academically in school (Nyadanu et al. 2015:266). Yet, at a higher educational level where there are different socio-cultural practices, existing educational policies and varied modes of lecturing, positive relationships might not be automatic (Nyadanu et al. 2015:273). There is, therefore, a need for the teachers or nurse educators to instil in the nursing students a sense of good relationships to ensure quality of health care delivery (Chan et al. 2014:386).

The use of the English language was another perceived barrier to understanding topics discussed in class by 69% (*n* = 69) of the respondents. In KwaZulu-Natal province, especially in the rural areas, students are taught in isiZulu in high school. The consequence is that many of them cannot read or write English adequately, which affects their ability to achieve academic success and their reading-to-learn ability in higher education (Pretorius 2005:536). It can be assumed that many students may first read the question, then translate it into the home language, and then choose the correct answer, eventually wasting precious time.

### Learning facilities

Six items helped to determine the student nurses’ perceptions of factors relating to the learning facilities that influenced their academic performance (Table 2). Although 77% (*n* = 77) of the respondents agreed that availability of study space (classroom and accommodation) supported their academic achievement, 37% (*n* = 37) and 40% (*n* = 40) of them felt that their school library and their school computer laboratory were not good predictors of improved academic performance.

**TABLE 2:** Student nurses’ perceptions of factors relating to learning facilities that influenced their academic performance.

Factors relating to learning facilities that influenced the students’ academic performance	Disagree		Neutral		Agree	
	n	%	n	%	n	%
The availability of study space (classroom and accommodation) supports my academic achievement in the school.	77	77	10	10	11	11
The school library is well stocked with relevant reading materials for learning to improve my performance academically.	44	44	17	17	37	37
The computer laboratory is adequately stocked with computers for my learning.	42	42	16	16	40	40
The entry qualification (matric results) of students is a predictor of good or bad academic performance in the nursing training college.	40	40	32	32	17	17
Availability of qualified nurse educators ensures positive learning outcomes in terms of student achievements.	93	93	6	6	1	1
The teaching strategies used by the nurse educators help me to understand the topic(s) under discussion during lessons which also improve my academic performance.	86	86	9	9	3	3

*n*, number of respondents.

The other learning facilities identified to be influencing the students’ academic performance were reported to be the availability of qualified nurse educators and the teaching strategies used by their nurse educators which helped them to understand the topics discussed in class by 93% (*n* = 93) and 86% (*n* = 86) of the respondents, respectively.

The entry qualification (high school results) was identified by 50% (*n* = 50) of the respondents as a predictor of good or bad academic performance. Thus, admission boards use prior academic performance to select students for admission (Kyoshaba 2009:26). An open challenge for nursing training including universities is therefore to recruit students who have previously demonstrated superior scholastic aptitudes (Dante, Petrucci & Lancia 2012:47; Lancia et al. 2013:1502).

### Proper guidance

In order to determine the student nurses’ perceptions of factors relating to the proper guidance that influenced their academic performance, four statements were used (Table 3). The results show that 91% (*n* = 91) of the respondents supported the view that parental involvement in the education of their children is a good predictor of better academic performance. When children are surrounded by caring, capable parents and are able to enjoy nurturing and moderate competitive kinship, a foundation for literacy is built with no difficulty (Zang & Carrasquillo 1995, cited by Vahedi & Nikdel 2011:333). Parents can also encourage their children to get high grades.

**TABLE 3:** Student nurses’ perceptions of factors relating to proper guidance that influenced their academic performance.

Factors relating to proper guidance that influenced the students’ academic performance	Disagree		Neutral		Agree	
	n	%	n	%	n	%
Parents’ involvement in the education of their children is a good predictor of better academic performance of students.	91	91	7	7	2	2
Students whose parents are literate have greater advantage in their academic performance than those whose parents are illiterate.	45	45	20	20	22	22
Academic support services offered by teachers help improve my academic performance.	88	88	10	10	1	1
Adequate support given at the clinical area during clinical attachment improve my practical skills and academic achievements.	88	88	5	5	4	4

*n*, number of respondents.

Having literate parents was also an advantage in academic performance according to 55% (*n* = 55) of the respondents. This may be because educated parents are well aware of the challenges in education and thus provide their children with moral strength to perform at their best. By extension, illiterate parents might not foster a study culture in their children because they themselves have not attended school to acquire the awareness of such an issue.

Additionally, 84% (*n* = 84) of the respondents agreed that academic support services offered by teachers help to improve their academic performance. Similarly, 88% (*n* = 88) of them reported that adequate support received at the clinical area during attachment improved their practical skills and academic achievements.

### Family stress

In order to determine the student nurses’ perceptions of factors relating to family stress that influenced their academic performance, four statements were used (Table 4). The results show that parental socio-economic background has a greater impact on students’ academic performance. Just over half of the respondents, 51% (*n* = 51) agreed that distance from home to school alters their academic performance. Similarly, 47% (*n* = 47) of the respondents agreed that a poor family background resulted in lower in their academic performance than a rich family background. A rationale to that could be that students from poor family backgrounds engage in menial jobs to contribute to the financial upkeep of the household which affects their academic performance. In contrast, being a female student and being advanced in age were reported to be not significant in academic performance. Only 18% (*n* = 18) and 29% (*n* = 29) of the respondents, respectively, found gender and age to be influencers of their academic performance.

**TABLE 4:** Student nurses’ perceptions of factors relating to family stress that influenced their academic performance.

Factors relating to family stress that influenced the students’ academic performance	Disagree		Neutral		Agree	
	n	%	n	%	n	%
Students from poor family background have low scores in their academic performance compared to their rich family counterparts.	47	47	19	19	34	34
Far distance residence to school affects the student’s academic performance.	51	51	24	24	24	24
Female students perform better than male students do academically.	18	18	11	11	68	68
Advanced in age has an impact on student nurses academic achievement.	29	29	19	19	51	51

*n*, number of respondents.

## Discussion

In this study, matric results (entry qualifications) were good predictors of one’s academic performance (good or bad) at the nursing training college. Similarly, Kyoshaba (2009:27) indicated that high school grades are the best predictors of good academic performance in higher education. This result is also in line with the contention by McCarey et al. (2007) that students with higher-level entry qualifications perform consistently better than those with lower-level qualifications in higher education. Moreover, Lancia et al. (2013:1501) found that students who failed in nursing education were those who had lowest grades associated with their upper-secondary diploma coursework. Ali and Naylor (2009:161) reported that Pakistani students with better academic performance in secondary school were found to have a significantly better performance in yearly nursing licensing examinations. This implies that upper-secondary diploma coursework grades are a parameter that should receive particular consideration, as they have higher predictive value for the academic success of nursing students. Therefore, secondary school academic performance should be the traditional admission pathway to tertiary education (Pitt et al. 2012:903).

The respondents of this study disagreed with the perception that female students perform better than male students academically. Contrary to this finding, however, some studies have shown that females outscore males academically (Al-Hussami et al. 2011; McCarey et al. 2007; Shulruf et al. 2011). Similarly, it is found that women perform better than men in colleges and universities (Hyde & Kling 2000, cited in UK Essays 2013). Ali and Naylor’s (2009:162) study revealed that female diploma nursing students in Pakistan performed significantly better than male students. This could imply that the poor academic performance of male students could be because of loss of concentration in class during lessons when they sit next to their female counterparts (Chan et al. 2014:378).

The results show that the use of ICT has a positive effect on the academic performance of student nurses. Communication through phone, emails and electronic discussion boards makes it easy to transmit information between the teacher and the learner. Similarly, Zawaduk et al. (2014:215) noted that ICT facilitates transmission of information between the teacher and the learner. For Kernan, Bogart and Wheat (2011:425), ICT is positively related to the performance of the student in open learning. Therefore, cellular phones or networks as technological resources should be considered an integral part of any education programme, because they are acceptable and used by the majority of South Africans (Department of Health 2012:31). However, internet usage and surfing are one of the factors affecting students’ academic achievements (UK Essays 2013). The risk of being distracted by social media is still high.

Findings from the study revealed that a good and supportive relationship between nurse educators and students fostered better academic performance of students. In line with these findings, Chan et al. (2014:386) and Myers and Pianta (2008) indicate that the teacher–student relationship is fundamental to the healthy development of students and their overall academic achievements in schools. Likewise, Ewnetu and Fisseha (2008) asserted that positive relationships are associated with better achievement and negative ones are related to a downward trend in academic achievement. Nyadanu et al. (2015:266) note further that the role of teachers in promoting the academic work of students is essential, especially in professional training such as training for nursing where nurses are trained to handle human life. Their role can be extended to the provision of the necessary assistance and psychological support for students from different family backgrounds to overcome their emotional problems and improve academic performance (Ushie et al. 2012:185).

Furthermore, in this study the majority of the respondents, 84% (*n* = 84), concurred with the suggestion that academic support services offered by teachers helped to improve their academic performance, and 88% (*n* = 88) of the respondents were of the view that adequate support received at the clinical area during attachment improved their practical skills and academic achievements. Similar to these findings, Cooper (2010:23) noted that one strategy for increasing student persistence and both academic and clinical performance outcomes lies in the area of student support services. These types of services are a standard feature at most higher education institutions and therefore play a role in promoting successful outcomes for nursing college students. Nursing education is strongly related to theory and practice applied in a realistic environment with faculty guidance and support. Therefore, the atmosphere should be welcoming and the environment should be committed to supporting the students’ learning (Courtney-Pratt et al. 2011).

Overall, positive learning outcomes in terms of student achievements are ensured through the availability of properly trained teachers, suitable learning resources and an appropriate learning environment (Bariso 2008:114). In fact, the falling standard of academic achievement is attributable to teachers’ own verbal reinforcement strategy and level of education (Gegbe & Koroma 2014:241). Poor academic performance of students is reflective of the attitude of some teachers to their job, characterised by behaviour such as poor attention to lessons, being late for work, unpleasant comments about students’ performance (that damage their ego) and poor teaching methods (Ulug, Ozden & Eryilmaz 2011). In addition, teaching strategies that have stronger focus on evidence-based and student-centred learning enhance academic performance and clinical competence of students. For instance, problem-based learning and case-based learning put a stronger focus on evidence-based and student-centred learning that will potentially enhance academic performance and clinical competence of students (Murphy et al. 2010:e147; Stanley & Dougherty 2010:378). However, traditional teaching approaches that focus mainly on information-giving activities in the classroom, firmly controlled by the teacher, do not promote realistic academic performance (Chilemba & Bruce 2015:55; Van Zyl 2014:20).

## Limitations

The first limitation of this study is related to the setting. The investigation was conducted in one school of nursing in South Africa and involved a sample population of 120, with only 100 participants responding to the research questionnaires. As a result, the findings of this study cannot be generalised. Moreover, the study was unable to capture the factors related to the role of the students in improving their own academic performance. Finally, a qualitative enquiry would have given deeper information about the factors influencing the students’ academic performance.

## Conclusion

It emerged from the study that several factors were perceived as positive and negative influencers of students’ academic performance. Factors perceived as contributing to poor academic performance of students included poor family background, peer group influence and use of English language for classroom instruction. In addition, poor matric results and distance to school were also found to be responsible for poor academic performance. However, respondents also identified some factors that could improve the academic performance of students. Chief among them were parental involvement in education; good nurse educator–student relationship; academic support services offered by teachers; use of information and communication technologies for learning, coupled with adequate learning facilities (library, computer laboratory and classroom space); availability of qualified nurse educators; and utilisation of effective and evidence-based teaching strategies.
